# Heterogeneity of the MDCK Cell Line and Its Applicability for Influenza Virus Research

**DOI:** 10.1371/journal.pone.0075014

**Published:** 2013-09-13

**Authors:** Vladimir Y. Lugovtsev, Darya Melnyk, Jerry P. Weir

**Affiliations:** Division of Viral Products, Center for Biologics Evaluation and Research, Food and Drug Administration, Bethesda, Maryland, United States of America; Centre of Influenza Research, The University of Hong Kong, Hong Kong

## Abstract

Single-cell clones have been established from the MDCK cell line, characterized for their morphology and evaluated for their suitability for influenza virus research. Three discrete cell morphotypes were identified using light microscopy. Besides morphological features, the cell types can be distinguished by the level of expression of surface glycans recognized by peanut agglutinin (PNA). All clones were susceptible to infection by influenza viruses of different subtypes of influenza A virus (H1N1, H1N1pdm09, H3N2, H5N1) and influenza B virus, and all possessed on their surface terminally sialylated glycans with both types of glycosidic linkage (α2–3 and α2–6). The Type-1 cell lines were able to support a multicycle replication of influenza A and B viruses without help of an exogenous trypsin. In contrast, cell lines exhibiting Type-2 morphology were unable to support multicycle replication of influenza A viruses without trypsin supplementation. Western blot analysis of the hemagglutinin of H1N1 strains demonstrated that Type-2 cells were deficient in production of proteolytically activated hemagglutinin (no cleavage between HA1/HA2 was observed). HA1/HA2 cleavage of influenza B viruses in the Type-2 cells was also significantly impaired, but not completely abrogated, producing sufficient amount of activated HA to support efficient virus replication without trypsin. In contrast, all clones of Type-1 cells were able to produce proteolytically activated hemagglutinin of influenza A and B viruses. However, the growth kinetics and plaque size of influenza A viruses varied significantly in different clones. Influenza B virus also showed different plaque size, with the biggest plaque formation in the Type-2 cells, although the growth kinetics and peak infectivity titers were similar in all clones. Taken together, the study demonstrates that the population of original MDCK cells is represented by various types of cells that differ in their capacities to support replication of influenza A and B viruses.

## Introduction

MDCK (Madin-Darby canine kidney) cell line was derived in 1958 by S.H. Madin and N.B. Darby from a kidney of a normal cocker spaniel [1], [2], using similar methodology as described for other two kidney cell lines of bovine and ovine origin [3], [4]. Soon thereafter, the first report of the susceptibility of this cell line to virus infection was published by Green [5]. Gaush and co-workers characterized MDCK cells by their growth, immunologic, and cytogenetic properties, as well as their susceptibility to several viruses [6]. Since then, the MDCK cell line has been extensively used as a model for studying the differentiated epithelial cells and renal ion-transporting mechanisms in epithelia [7]–[24]. Due to its high susceptibility to various influenza viruses the MDCK cell line remains the most widely used cell line in influenza virus research [25]–[42]. In addition, it was found that human influenza viruses isolated and propagated in MDCK retain their original antigenic properties, that makes this cell line a suitable substrate for selection of influenza vaccine strain candidates and a platform for vaccine development [43]–[47].

From the very beginning, it was noted that MDCK cultures contained a heterogeneous cell population, and analysis of the MDCK cell lines from different laboratories revealed the variability in the modal number of chromosomes, morphology, and other characteristics. Cloning of the original MDCK cell culture resulted in the selection of cell lines that could be distinguished by their morphological, electro-physiological, and biochemical properties [6], [7], [24], [48]–[63].

In this study, we have investigated the heterogeneity of the MDCK cell line in the context of the applicability of cell clones with various properties to influenza virus research. We selected cell lines representing at least three major cell types with morphological and physiological characteristics similar to those described earlier by other researchers, and characterized these clones for their susceptibility to influenza viruses, expression of the influenza virus receptors, ability to produce proteolytically activated viral hemagglutinin, and practical applicability for virology techniques.

## Materials and Methods

### Cell Lines

MDCK cell line (NBL-2, ATCC-CCL-34, Lot 4398972, passage 56) was obtained from ATCC (Manassas, VA) and carried for another 20 passages before cloning. Cloning was performed by limiting dilutions in 96-well plates, using a suspension of cells with a calculated concentration 1 cell per ml (distributing 0.1 ml per well). Clones originated from a single cell were propagated in EMEM (Lonza, Cat#12-611F) with 10% FBS (HyClone, Cat# SH30910) and non-essential amino acids (Gibco, Cat# 11140-050), and after second passage aliquots of each clone were frozen in liquid nitrogen in the cryo-protective freezing medium “Recovery™ Cell Culture Freezing Medium” (Invitrogen/Gibco Cat # 12648-010). Cell cultures of clones were maintained for up to 25 passages under standard conditions (at 37°C with 5% CO_2_).

### Viruses

All viruses used in this study were obtained from the CBER Influenza Virus Depository (Division of Viral Products, OVRR/CBER/FDA). The working virus stocks were prepared by propagation in chicken embryonated eggs (allantoic fluid). The following influenza viruses were used in this study: influenza A viruses A/Brisbane/59/2007 IVR-148 (H1N1, seasonal); A/California/7/2009 X-179A (H1N1pdm09), A/Christchurch/16/2010 (H1N1pdm09), A/Uruguay/716/2007 X-175C (H3N2), rg-A/Vietnam/1203/2004 (H5N1, low-pathogenic) and influenza B viruses (B/Hubei-Wujiagang/158/2009, and B/Victoria/504/2000). The two variants of B/Victoria/504/2000, which were generated by the reverse genetics and differ from each other by three amino acid substitutions in the receptor-binding pocket of the viral hemagglutinin and thus, preferentially bind to carbohydrates terminally sialylated either via α2–6 glycosidic linkage, or α2–3, have been described earlier [64].

### Cell Morphology

Morphological characterization of the selected cloned cell lines was based on phase-contrast light microscopy of the confluent cell monolayer at different passages (up to 15–25 passages, with splitting ratio 1∶10). Cell monolayers were evaluated for the shape and relative size of the cells, the visibility of the cell borders (intercellular space) and nuclei, the presence of large extracellular liquid-filled structures (hemicysts, mostly referred to as “domes” [65], [66], and the appearance of abnormally large (“giant”) multinucleated cells. Cell cultures were analyzed using an inverted microscope (Zeiss Axiovert 40 CFL with 5×, 10×, and 20× objectives) and digitally visualized using software AxioVision 4.8.2.

### Evaluation of the Virus Growth Kinetics in Cell Clones

Confluent cell monolayers (T75 flasks, Corning Cat# 430641) were rinsed with PBS (pH 7.2) and inoculated with the virus at a low multiplicity of infection (MOI = 1∶10^6^). After incubation for 2 hours at 37°C and 5% CO_2_, the cells were rinsed twice with PBS to remove the unbound virus and incubated in EMEM without FBS at 33°C, 5% CO_2_, for 3–8 days (depending on cell viability associated with cytopathic effects (CPE). Aliquots of infected culture medium were collected daily, stored frozen at −70°C, and evaluated for virus accumulation by titration of hemagglutination and infectivity (TCID_50_). If not stated otherwise, virus growth kinetics was evaluated in cell cultures maintained without exogenous trypsin in the medium. The effect of exogenous trypsin on the growth of H1N1 virus was evaluated in an independent experiment with the final trypsin concentration in the maintenance culture medium at 1.0 µg/mL.

### Hemagglutination Assay

The hemagglutination assay was performed using 0.5% chicken red blood cells (in PBS, pH 7.2) in round-bottom 96-well plated (Corning Cat# 3797) by a standard technique [67], [68].

### Virus Infectivity Titration, TCID_50_

TCID_50_ (50% tissue culture infectious dose) titers were determined using the original MDCK cells (ATCC-CCL-34, NBL-2, Lot 4398972). Cells grown to the confluence in flat-bottom 96-well plates (Corning Cat#3585) were washed with PBS (pH 7.2) and inoculated with serial 10-fold dilutions of the virus sample (diluted in full EMEM medium but without FBS). Inoculated cells were incubated at 33°C, in an atmosphere containing 5% CO_2_ for seven days. Cell infection in a given well was determined by CPE, which is usually associated with cell death and complete destruction of the monolayer. TCID_50_ titers were calculated by the method of Reed and Muench [69].

### Evaluation of Permissiveness of the MDCK Cell Clones to Influenza Virus Infection

Permissiveness of the MDCK cell clones to various influenza viruses was evaluated by comparative TCID_50_ titration of the egg-derived reference viruses in the corresponding cloned cell lines. Cells of each of the clones were grown to the confluence in 96-well plates and infected with serial 10-fold dilutions of the corresponding virus. In addition, the virus titer in each clone was determined by plaque assay with calculation of plaque-forming infectious units (PFU).

### Plaque Assay

Cells grown to confluence in 12-well plates (Corning, Cat#3512) were washed with PBS (pH 7.2) and inoculated with serial 10-fold dilutions of the virus. After 1 h at 37°C, the cells were washed with PBS to remove the unbound virus, and overlaid by MEM (Lonza, Cat#12–668E) containing 0.75% agarose (Sigma, Cat# 15510-027) without FBS. Plaque assays with influenza A viruses were performed using overlaying agarose-containing medium either without or with TPCK-Trypsin at final concentration 1.5 µg/mL (Worthington, TRTVMF, 3750). Plaque assays with influenza B viruses were performed without exogenous trypsin if not stated otherwise. Infected cells were incubated at 33°C for 96 hours (in an atmosphere with 5% CO_2_), fixed with cold 96% ethanol, and stained with 1% crystal violet (Sigma, Cat#HT90132).

### Detection of the Sialylated Glycans with α2–3 or α2–6 Glycosidic Linkage on the Cell Surface

Detection of the of influenza virus receptors on the cell surface, the terminally sialylated carbohydrates with α2–3 or α2–6 glycosidic linkage, was performed by flow cytometry. Sialic acid moieties with α2–3 glycosidic linkage were detected by biotinylated lectin from *Maackia amurensis* (MAL-II; Vector Laboratories, Inc., Cat# B-1265) in combination with Streptavidin conjugated with R-Phycoerythrin (Vector Laboratories, Inc., Cat# SA-5207). Sialic acid moieties linked *via* α2–6 were detected by FITC-labeled SNA (*Sambucus nigra* (Elderberry) Bark Lectin; Vector Laboratories, Inc. Cat# FL-1301). Cells were prepared as described previously [32] and treated with lectins in accordance to the manufacturer’s instruction. Briefly, cells grown to confluence in T225 flasks (Corning, Cat# 431082) were released from flask surface by standard procedure using trypsin-versene mixture (Lonza Cat#17–161E), washed once with EMEM with 10% FBS, resuspended in 40 mL of the same medium, and incubated at 37°C for 1 hour to restore the trypsin-digested receptors. After precipitation by low-speed centrifugation, the cells were resuspended in PBS (pH 7.2) to a final concentration 10^6^ cells/mL. After treatment with lectins, the cells were washed twice with PBS (pH 7.2) and resuspended in 0.5 mL PBS containing 1 mM EDTA. The prepared samples were analyzed on “BD™ LSR II” flow cytometer using FACSDiva v.6.2 software (BD Biosciences).

### Detection of PNA-specific Ligands (Glycans Terminated by Galactose) on the Cell Surface

Detection of the cell-surface O-glycans terminated by non-sialylated galactose [Gal-β(1–3)-GalNAcα1-R], the specific ligand recognized by a lectin peanut agglutinin (PNA), was performed by flow cytometry using the same procedure as for the detection of terminally sialylated glycans described above. FITC-labeled PNA was used in accordance with the manufacturer’s instruction (Vector Laboratories, Inc. Cat# FL-1071).

### Characterization of the HA1/HA2 Cleavage Profile

The HA1/HA2 cleavage profile of the HA of viruses grown in different cell clones was evaluated by SDS-PAGE (under reducing conditions) followed by Western blot with anti-HA1 or anti-HA2 antibodies. Virus particles produced in different cell clones were concentrated and purified through 29%–49% sucrose gradient, treated by 0.5% Triton X-100 for 1 hour at room temperature, and applied for SDS-PAGE separation using NuPAGE® 10% Bis-Tris Gel (Invitrogen, Cat# NP0302BOX). Proteins were transferred to the PVDF membrane using iBlot® gel transfer system (Invitrogen, Cat# IB1001; Cat# IB4010-02), followed by protein visualization using WesternBreeze® Chromogenic Immunodetection system (Invitrogen, Cat# WB7103, or Cat# WB7105). Molecular weight of the proteins separated in gel was estimated in comparison with the standard protein marker “MagicMark™ XP Western Standard” (Invitrogen, Cat# LC5602). The anti-HA2 mouse monoclonal antibodies (MyBioSource, Cat# MBS430056) were used as primary antibodies for the analysis of influenza B viruses. Rabbit polyclonal antibodies specific to HA1 or HA2 of H1N1 influenza A viruses were kindly provided by Dr. Carol Weiss and Dr. Wei Wang (DVP/OVRR/CBER/FDA).

### Statistics

A two-tailed Student’s *t* test was used to determine statistically significant differences. Differences were considered significant if the *P* value was <0.05 (if not stated otherwise).

## Results

### Cell Morphology of Cloned MDCK Cell Lines

**Figure 1:**
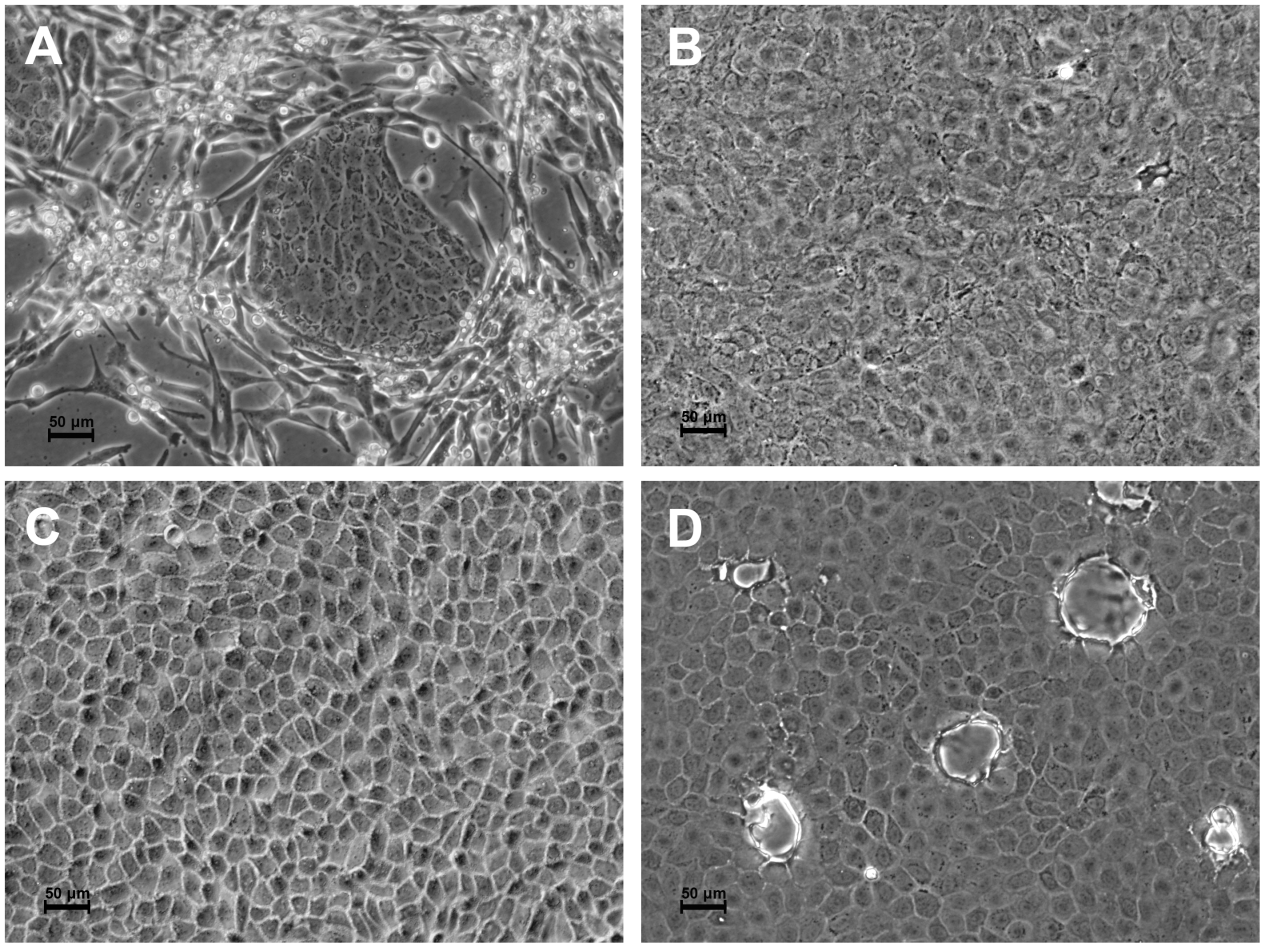
Microphotographs of the parental MDCK and cloned cell lines. **A.**Original MDCK (ATCC-CCL-34, NBL-2; +7 passages). **B.** Type-1 cells (clone 4F2; +5 passages after cloning): gentle polymorphic cells with a visible nucleus and very fine almost indistinguishable intercellular space, cells often develop large cytoplasmic area. **C.** Type-2 cells (clone 3D7; +4 passages after cloning): polygonal cells with almost invisible nuclei; the intercellular space is well developed, and contributes to a specific a mosaic-like appearance of the monolayer. **D.** Formation of the numerous liquid-filled structures, “domes”, visible as rounded refracting blisters on the monolayer of the Type-3 cells (clone 3E11; +11 passages after cloning).

MDCK cells, originally obtained from the American Type Culture Collection (ATCC-CCL-34), appear to contain a variety of heterogeneous cell types, including a sparse network of fibroblast-like cells (spindle-like elongated cells) and islands of epithelial-like cells (Fig. 1A). From this cell line ten individual MDCK cell lines were isolated, cloned by limiting dilution, and characterized. Systematic monitoring of the obtained cell lines revealed that individual cell clones exhibited different morphological properties (Figure 1), and most could be grouped into two distinct morphotypes similar to the previously described Type-1 and Type-2 cells [24], [49], [51], [52], [57], [61]. All of the cloned MDCK cell lines stably maintained their morphological phenotype for at least 25 consecutive passages (the current period of observation).

The Type-1 cells (1B11, 2C10, 2F8, 3D10, 4B7, 4F2, 5C9) appeared as a thin transparent monolayer of flattened polymorphic cells (oval, elongated, spindle-like) with a clearly visible nucleus and very fine almost indistinguishable cell borders (Fig. 1B). Cells of this type often demonstrated a tendency to form an enlarged cytoplasm. Occasionally, very large multinucleated cells (“giant” cells), located individually or as small clusters, were observed in apparently normal cell monolayers (data not shown). Under standard growth conditions, the formation of domes was not observed.

Cells of Type-2 morphology (3D7, 4D2) appeared as coarse, thick monolayers (with low transparency) of relatively small cells (small surface area) with an almost invisible nucleus and with very well distinguished often grainy rough intercellular space. The cells had a characteristic angled polygonal shape (squared, rhomboid, or hexagonal) resulting in a monolayer resembling a mosaic-like surface (Fig. 1C). Cells of this type did not form liquid-filled extracellular domes, and giant cells were not observed.

One clone, 3E11, was separated and designated as an individual group, being distinguished from the other cell lines by the ability to form numerous liquid-filled extracellular structures under normal conditions (i.e. without induction by chemical or physical treatment) (Fig. 1D). By appearance the liquid filled structures resembled large rounded or oval cysts involving several adjacent cells, also described in the literature as “domes” [65], [66], [70]. Microscopic monitoring of the dome-forming clone, 3E11, showed that its morphology was very similar to that of the Type-2 cells (mosaic monolayer of angled polygonal cells with bright-phase borders and a hardly visible nucleus). At the same time, other properties described below in this study, such as the level of expression of PNA ligand, proteolytic potency ensuring HA1/HA2 cleavage of virus hemagglutinin, and the support of multicycle replication of influenza A viruses without exogenous trypsin, make this clone closer to Type-1 cells. Therefore, taking into account all evaluated phenotypic properties, this cell clone could not be classified as an authentic representative of Type-1 or Type-2 cells and was segregated as a representative of another cell type (Type-3; “dome-forming” cells).

### Detection of PNA-receptors (Glycans Terminated by Galactose) on the Cell Surface

It has been shown earlier that selective recognition and binding of PNA to the non-sialylated terminal galactose moiety of the O-glycans [Gal-β(1–3)-GalNAc-R], can be used as a marker for identification of specific renal epithelial cells, representing differently specialized subpopulations of cells [51], [53], [71]–[77].

**Figure 2:**
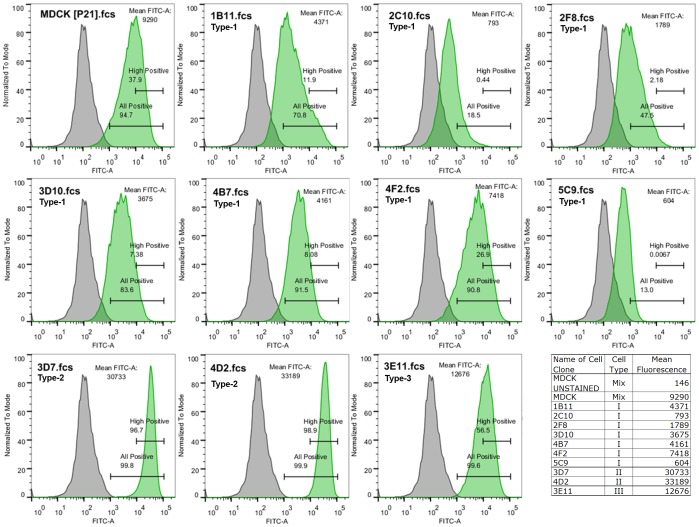
Evaluation of the level of the surface expression of the PNA-specific carbohydrates. The level of cell surface expression of PNA-specific glycans was evaluated by flow cytometry using FITC-labeled PNA. Each panel shows intensity of fluorescence (X axis) in relation to the normalized cell counts (Y axis) of cells treated by the fluorescent probe (green peak), in comparison with an unstained control cells (gray peak). Gates indicate the percentage of “All-Positive” cells, and “High-Positive” cell. The mean fluorescence is shown in the upper right corner of each panel and summarized in the table included in the right corner of the figure. Data from one representative experiment.

The level of cell surface expression of PNA-specific glycans was evaluated using flow cytometry by binding with a specific FITC-labeled PNA (Fig. 2). The data obtained in this analysis demonstrated that the Type-1 and Type-2 cells had different patterns of the expression of carbohydrates recognized by PNA (Fig. 2). Thus, both clones of Type-2 cell, 3D7 and 4D2, uniformly showed a high level of PNA binding by all cells in population. The PNA-binding by Type-2 cells was substantially higher than it was by cells of all other clones (Fig. 2; Fig. S3). Therefore, the range of their intensity of fluorescence was used as the criterion for “High-Positive” PNA binding. The proportion of the PNA-positive cells in the populations of the Type-1 cell clones varied significantly between clones, from very low (2C10 and 5C9) to relatively high (4B7 and 4F2), but the representation of the “High-Positive” cells was low in all Type-1 clones. The dome-forming Type-3 cells (clone 3E11) showed intermediate level of PNA-binding if compared with Type-1 and Type-2 cells. Thus, the level of expression of the PNA-specific ligand can be used as a specific marker for the phenotypic characterization of populations of MDCK cells in culture.

### Detection of α2–3 or α2–6 Sialylated Glycans on the Cell Surface

**Figure 3:**
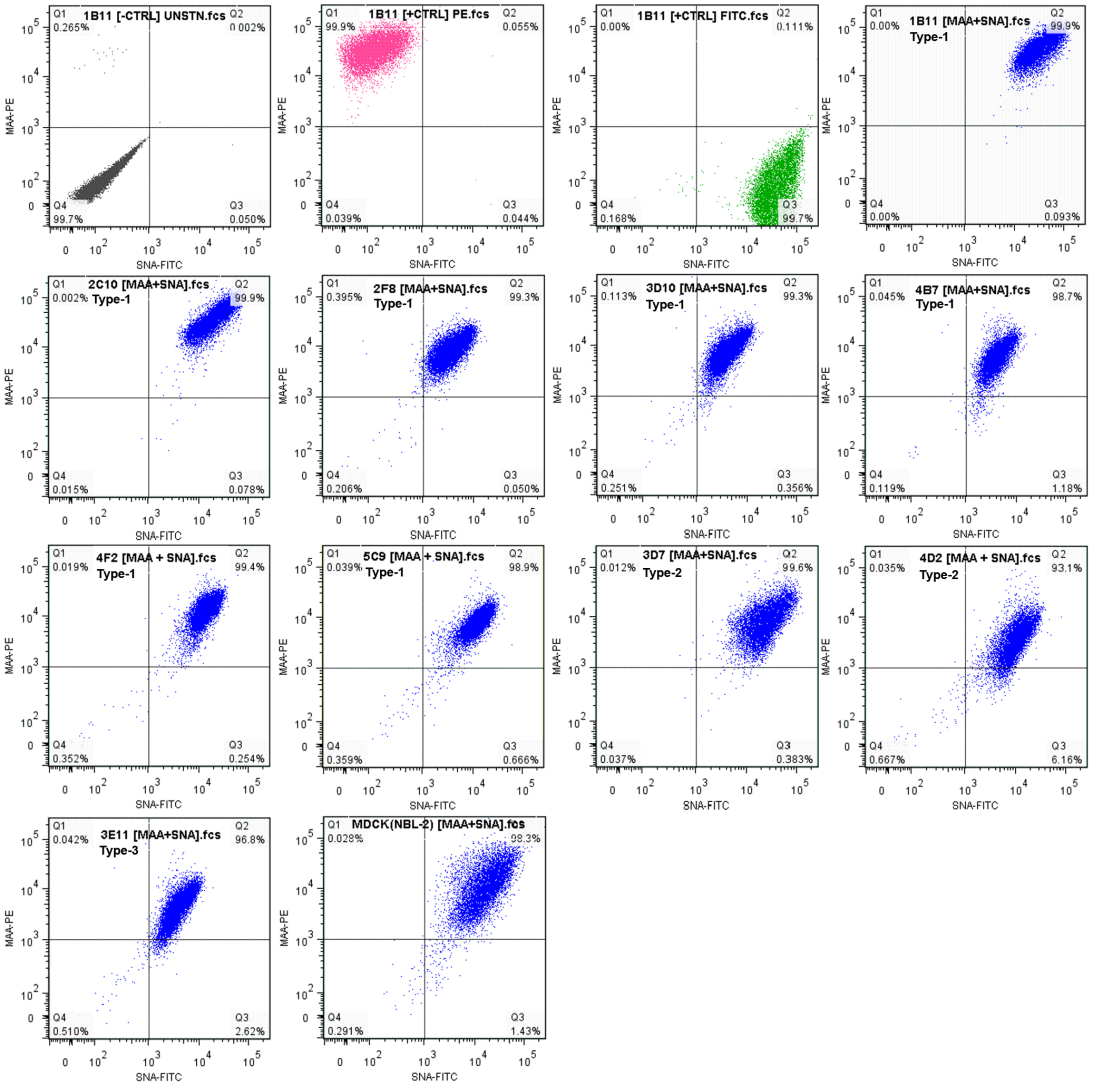
Detection of α2–3 or α2–6 sialylated glycans on the cell surface. Detection of the of influenza virus receptors on cell surface, the carbohydrates terminally sialylated via α2–3 or α2–6 glycosidic linkage, was performed by flow cytometry. Sialic acid moieties with α2–3 glycosidic linkage were detected by biotinylated lectin MAA from *Maackia Amurensis* (MAL-II) in combination with Streptavidin conjugated with R-Phycoerythrin (PE); glycans with α2–6 linkage were detected by FITC-labeled SNA (*Sambucus Nigra* (Elderberry) Bark Lectin). Sample preparation and procedure are described in Material and Methods. Quadrant Q1: MAA(α2–3)-positive cells; Quadrant Q2: MAA(α2–3)+SNA(α2–6)-positive (double-positive) cells; Quadrant Q3: SNA(α2–6)-positive cells; Quadrant Q4: unstained cells [control]. Data from one representative experiment.

The representation of the influenza virus receptors, terminally sialylated carbohydrates with α2–3 or α2–6 glycosidic linkage between *N*-acetylneuraminic acid and the galactose, on the surface of the selected cells, was investigated by flow cytometry using lectins, which bind selectively either α2–3 or α2–6 linked sialic acids. Sialic acid moieties with α2–3 glycosidic linkage were detected by MAA (lectin from *Maackia Amurensis*, MAL-II), whereas α2–6 linked sialic acids were detected by SNA (*Sambucus nigra* (Elderberry) Bark Lectin). This qualitative analysis revealed that cells of all clones have on their surface sialylated glycans with both types of glycosidic linkage (Fig. 3).

### Evaluation of Permissiveness of the Cloned Cell Cultures to Influenza Virus Infection

**Figure 4:**
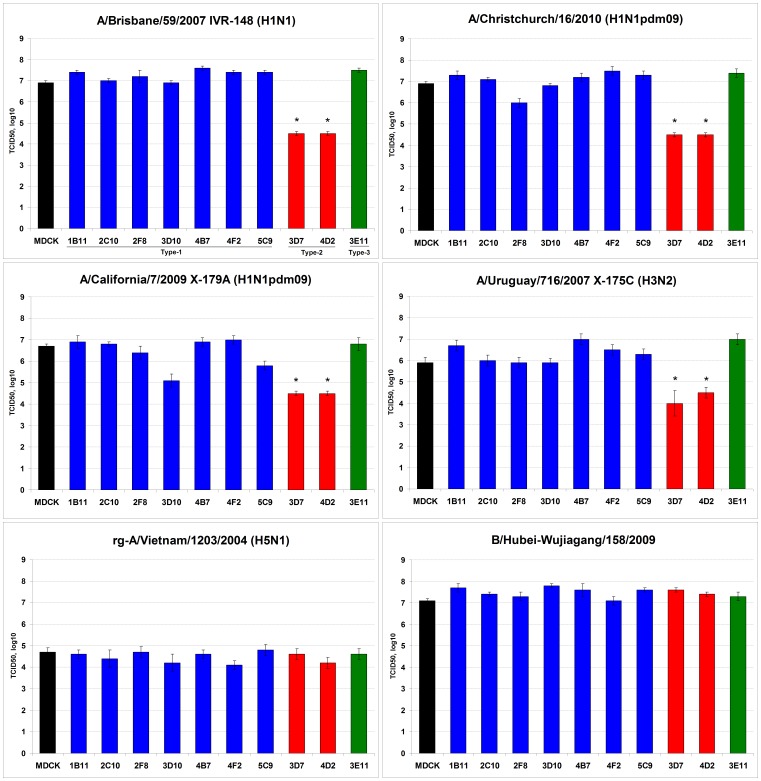
Infectivity titers of influenza virus strains in MDCK clones. Infectivity titers (TCID_50_, log_10_/0.1 ml) of reference viruses were determined using different cell clones and parental MDCK. Each panel represents infectivity titers for the indicated virus strain. Blue bars represent Type-1 cells; red bars – Type-2 cells; green bars – Type-3 cells. Values represent Mean ± SE (data from two independent experiments). Asterisk (*) indicates a significant difference (*P*<0.05) in comparison with the values in the parental MDCK cells (black bars).

In order to investigate the level of permissiveness of the cloned cell lines to various influenza viruses, the TCID_50_ titration of reference viruses was conducted in the original MDCK cells and each of the cloned MDCK cell lines. All of the MDCK cell lines were found to be susceptible to all influenza viruses used in the study, namely, IVR-148 (H1N1, seasonal), X-179A and A/Christchurch (H1N1pdm09), X-175C (H3N2), A/Vietnam (H5N1, low-pathogenic), and influenza B virus strain B/Hubei (Fig. 4). Influenza B virus and H5N1 virus showed indistinguishable titers in all cell lines, whereas virus yields of H1N1 and H3N2 viruses showed similar pattern of variability in titers in the corresponding cell clones, showing significantly lower titers in cell lines with Type-2 morphology (3D7 and 4D2), with an approximately 2 log10 decrease in virus titer compared to the corresponding values in other cell clones and parental MDCK (*P*<0.05). The results indicate that although all cell clones were susceptible to infection by various influenza viruses, not all clones were equivalent in supporting efficient multicycle replication of H1N1 and H3N2 viruses.

### Evaluation of Cell Clones by Plaque Size using Viruses with Preferential Binging of α2–3 or α2–6 Sialylated Glycans

**Figure 5:**
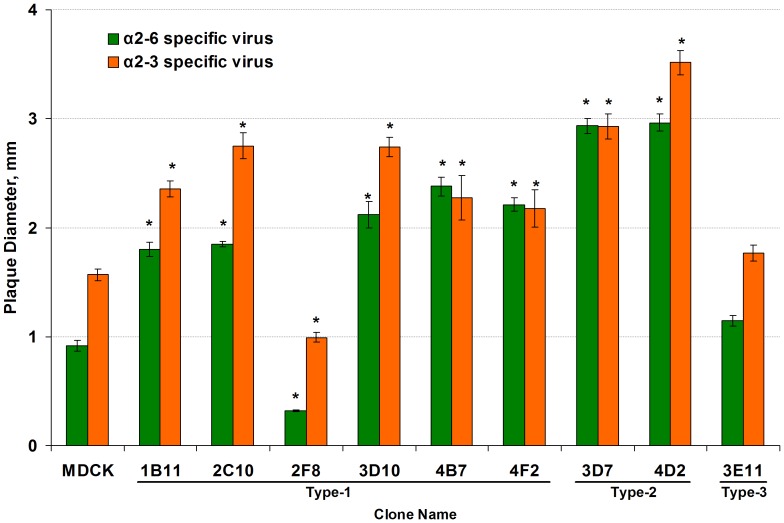
Plaque size of virus variants with α2–3 or α2–6 binding preferences in MDCK clones. Variants of influenza B virus strain B/Victoria/504/2000 with binding preferences for sialylated glycans with either α2–3 (orange bars) or α2–6 (green bars) glycosidic linkage were used as probes to investigate the cell clones for their support of replication of viruses with different receptor-binding properties. Bars represent the diameter (mm) of virus plaques in the corresponding cell clones 96 hours post infection, at 33°C. Values represent the Mean ± SE from two independent experiments. Asterisk (*) indicates a significant difference (*P*<0.01) in comparison with the corresponding values in the parental MDCK cells.

To evaluate if some of the cell clones could be more appropriate substrates for viruses with discrete preferences for either α2–3 or α2–6 terminally sialylated glycans, two influenza B virus variants that preferentially bind to either one or the other type of receptor were used as probes, and the efficiency of their replication was analyzed by their plaque-size phenotype. Though virus with preferences to the α2–3 sialylated glycans produced larger plaques in most of the cases than the virus with α2–6 specificity, both probing viruses showed similar trends in plaque-size phenotype in any given cell line. These data concurred with the observations obtained using MAA and SNA lectins, providing additional evidence that all cell clones have both α2–3 and α2–6 receptors in an amount sufficient to permit entry of viruses regardless of their receptor-binding preferences (Fig. 5). At the same time, individual cell lines could be distinguished by their ability to contribute to virus plaque size, a characteristic that was found to be unrelated to the virus receptor-binding preference. Two cell clones, 3D7 and 4D2 (both with Type-2 morphology), were the cell lines with the biggest plaques by each virus variant, whereas both viruses formed significantly smaller plaques in cell clone 2F8 (Type-1) and 3E11 (Type-3). In other clones, both viruses produced plaques of moderate size, though bigger than in the parental MDCK (*P*<0.01). Thus, all cell clones were similarly permissible for viruses with either α2–3 or α2–6 receptor-binding preference, but the size of the plaques was affected by some other physiological properties of the particular cell clones.

### Evaluation of Cell Clones in Plaque Assay with Different Types of Influenza Viruses

**Figure 6:**
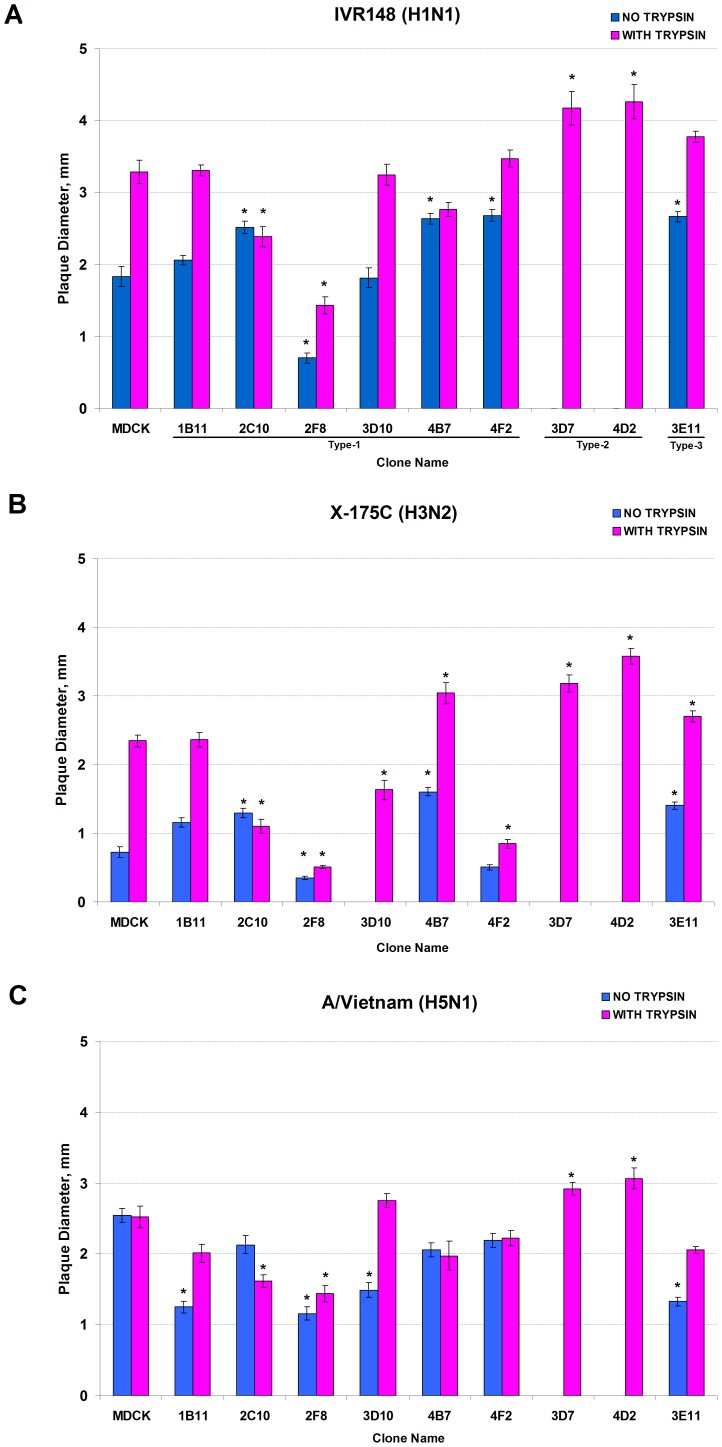
Plaque size of influenza A viruses in MDCK clones with and without trypsin. Panel A (upper panel): A/Brisbane/59/2007 IVR-148 (H1N1 seasonal). Panel B (middle panel): A/Uruguay/716/2007 X-175C (H3N2 seasonal). Panel C (lower panel): rg-A/Vietnam/1203/2004 (H5N1, low-pathogenic). Bars represent the diameter (mm) of virus plaques in parental MDCK and the corresponding cell clones 96 hours post infection, at 33°C. Blue bars represent plaque size without trypsin; pink bars represent plaque size in the presence of the trypsin in the overlaying agar media. Values represent the Mean ± SE from two independent experiments. Asterisk (*) indicates a significant difference (*P*<0.01) in comparison with the corresponding values in the parental MDCK cells.

The ability of the cell clones to support an efficient replication of viruses of different types and subtypes was also investigated by plaque assay (Fig. 6; Fig. S1). One cell clone, 5C9, was found unusable for plaque assay, being unable to survive in the agar-containing media. Plaque size phenotype for the remaining nine MDCK cell clones and the parental MDCK cells was evaluated in the presence or absence of the exogenous trypsin.

None of the influenza A viruses (H1N1 seasonal and H1N1pdm09, H3N2, and H5N1) were able to produce plaques on type-2 cells (3D7 and 4D2) in the absence of exogenous trypsin. On the other hand, supplementation of the media with the exogenous trypsin made these cell lines the optimal substrates in terms of producing the largest plaques for all tested viruses. The type-2 cell lines 3D7 and 4D2 were also the best substrates for plaque assay of influenza B viruses, producing the largest size plaques of all the cell lines regardless of the presence of trypsin. It was also observed during the course of these experiments that addition of trypsin had no effect on the plaque size of influenza B viruses in all cell clones used in this study (data not shown).

There were also differences in virus plaque size using the various type-1 cell lines. For example, all viruses produced very small plaques in the cell line 2F8, whereas plaques of the H3N2 virus (with or without trypsin) were substantially smaller using cell line 4F2, and plaque formation of influenza B virus was restricted in 3E11. Thus, the results indicated that virus plaque size differed significantly depending upon the MDCK cell clone used in the plaque assay (Table 1; Table S1).

**Table 1:** Phenotype of MDCK clones and their characterization as substrates for influenza A and B viruses.

	Cell phenotype				HA1/HA2 cleavage capability		Support virus replication without trypsin		Maximum HA Titer		Maximum virus accumulation, TCID_50_ log_10_/0.1 ml Mean ± SD		Plaque size, mm Mean ± SD		
MDCKClone	Morphotype	PNAbinding(Gal−)	MAAbinding(α2–3)	SNAbinding(α2–6)	H1N1IVR-148	B/Hubei	H1N1IVR-148	B/Hubei	H1N1IVR-148	B/Hubei	H1N1IVR-148	B/Hubei	H1N1Trypsin (−)	H1N1Trypsin (+)	B/HubeiTrypsin (−)
1B11	I	Low	+	+	+	+	+	+	32	64	6.4±0.3	7.0±0.1	2.1±0.5	3.3±0.5	1.2±0.4
2C10	I	Low	+	+	+	+	+	+	32	64	6.0±0.1	7.6±0.1	2.5±0.5	2.4±0.7	0.7±0.3
2F8	I	Low	+	+	+	+	+	+	2	64	4.8±0.1	7.0±0.1	0.7±0.2	1.4±0.4	0.8±0.3
3D10	I	Low	+	+	+	+	+	+	16	64	5.6±0.2	7.2±0.3	1.8±0.6	3.2±0.6	2.2±0.5
4B7	I	Low	+	+	+	+	+	+	32	64	5.5±0.4	7.6±0.1	2.6±0.5	2.8±0.5	1.2±0.5
4F2	I	Low	+	+	+	+	+	+	32	64	5.9±0.1	7.5±0.2	2.7±0.4	3.6±0.4	1.6±0.5
5C9	I	Low	+	+	+	+	+	+	2	64	5.0±0.4	7.5±0.1	*No data*	*No data*	*No data*
3D7	II	High	+	+	–	±	–	+	256+T	64	7.4±0.2+T	6.9±0.3	0	4.2±0.6	1.5±0.4
4D2	II	High	+	+	–	±	–	+	256+T	64	7.3±0.2+T	7.1±0.2	0	4.3±0.8	1.8±0.4
3E11	III	Medium	+	+	+	+	+	+	128	128	6.1±0.4	8.1±0.2	2.7±0.6	3.8±0.3	0.8±0.2
MDCK(parent)	*mix*	Medium	+	+	+	+	+	+	64	64	5.9±0.2	7.1±0.3	1.8±0.5	3.3±0.0	0.5±0.1

+T – Virus replication was observed only in the presence of exogenous trypsin in the culture media.

### Evaluation of the Virus Growth Kinetics in Cell Clones

The dynamic of virus accumulation in cell clones was evaluated by infection of confluent cell monolayer in the absence of exogenous trypsin at a low multiplicity of infection (MOI = 0.000001); this assay requires multiple cycles of virus replication and secondary infections. One influenza A virus (IVR-148, H1N1 seasonal) and one influenza B virus (B/Hubei-Wujiagang/158/2009) were selected as model viruses for the kinetics experiments.

**Figure 7:**
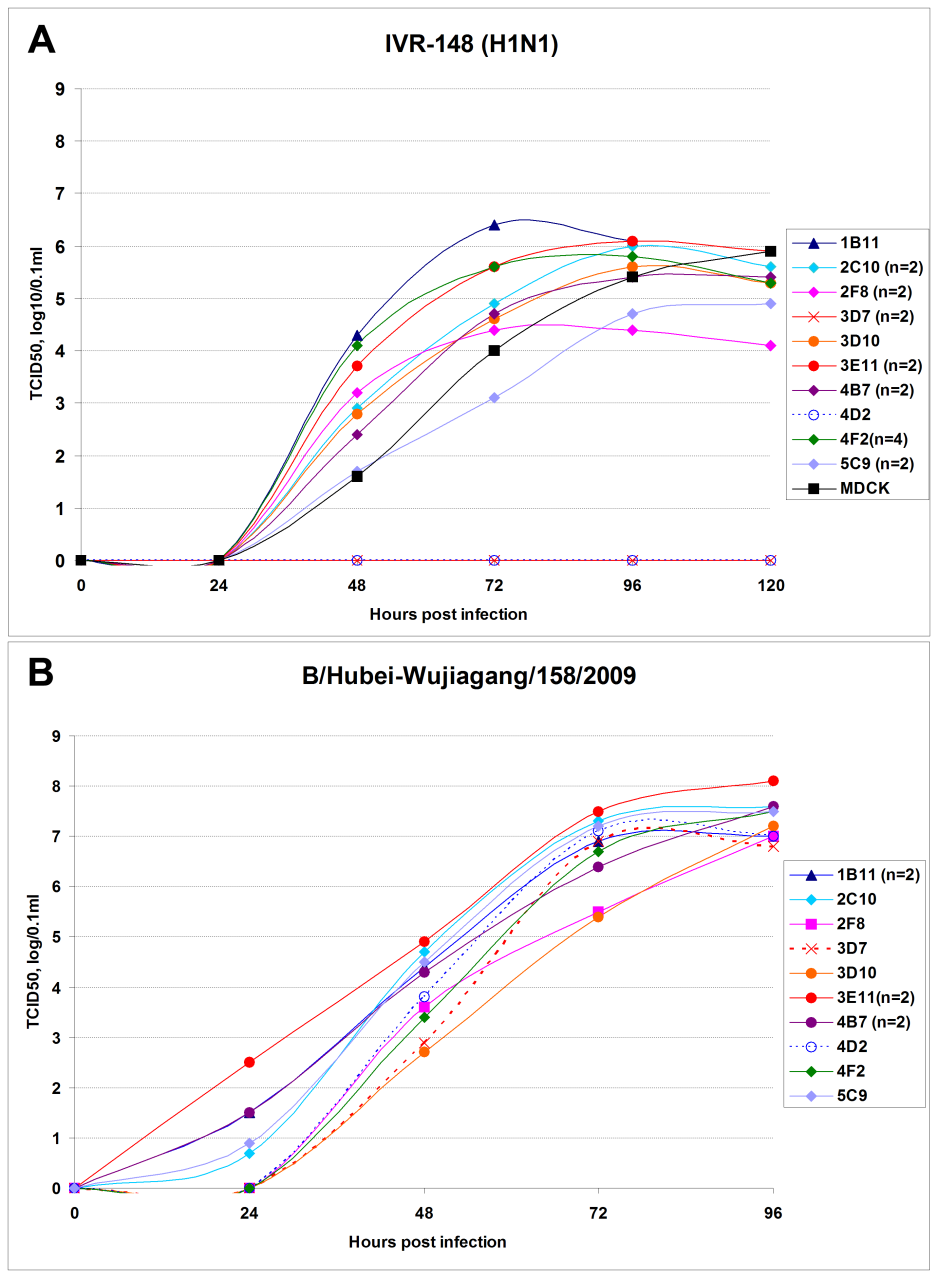
Growth kinetics of influenza viruses in MDCK clones (without exogenous trypsin). Growth kinetics of influenza A and B viruses in MDCK clones were determined by infectivity titration (TCID_50_, log_10_/0.1 ml) of the cell culture media samples collected at different time-points post infection. Culture of the corresponding MDCK clones (confluent cell monolayer) were infected at MOI 0.000001, and incubated at 33°C until complete destruction of the monolayer due to CPE. Panel A (upper panel): A/Brisbane/59/2007 IVR-148 (H1N1, seasonal). Panel B (lower panel): B/Hubei-Wujiagang/158/2009.

All cell clones supported efficient replication of influenza B virus, which accumulated to similarly high infectivity titers, reaching 10^7^–10^8^ TCID_50_ per 0.1 mL in all clones and resulted in complete destruction of the cell monolayer. In most instances, virus replication reached its peak around 72–96 hours post infection (Fig. 7B). The slower virus accumulation was observed in cell lines 2F8 and 3D10, but nevertheless, despite the slower infection kinetics, virus infectivity and hemagglutination titers reached similar levels as with other cell clones by 96 hours post infection. In most of the clones, the maximum titers of hemagglutination were 1∶64, with the exception of 3E11, where the higher titer (1∶128) was reproducibly observed.

More variation in virus growth dynamics were observed with H1N1 virus (Fig. 7A). Infection of cell lines 3D7 and 4D2 (Type-2 cells) did not lead to the efficient virus propagation in the absence of trypsin, similar to the results obtain in the plaque assay analysis in the absence of trypsin. The monolayer remained intact (no CPE) for up to 8 days post infection (period of observation), and neither infectious virus nor hemagglutinating activity was detected during that period. Dramatic differences were observed when the culture medium was supplemented with exogenous trypsin after cell infection. In the presence of trypsin, virus replication was already detectable by 24 hours post infection (by infectivity and hemagglutination), with the peak of infection at 72 hours (accompanied by a complete destruction of the monolayer) (Fig. S2). For both cell lines 3D7 and 4D2, supplemented with trypsin, the kinetics of virus replication was identical, and the highest infectivity and hemagglutination titers were observed, with TCID_50_ titers reaching 7.4 log_10_/0.1 mL and titers of hemagglutination 1∶256 (Table 1; Fig. S2).

Type-1 and Type-3 cell clones were able to support influenza A virus replication without addition of trypsin to the medium, though in some of the clones virus accumulation was significantly inhibited compared with the overall trends in other clones. The lowest virus yield was observed in cell lines 2F8 and 5C9, where the hemagglutinating activity hardly reached the limit of detection (and did not increase thereafter), and distinguishable CPE began to appear only by 96 hours post infection. Prolonged monitoring (144 hours post infection, data not shown) indicated that virus replication in both cell lines reached a plateau by 72–96 hours post infection, even though only about 50% of the monolayer destruction was observed by 144 hours post infection. Infectivity titration of time-point samples also indicated inefficient virus replication, with peak titers not to exceed 5.0 log_10_/0.1 mL (TCID_50_).

H1N1 virus kinetics in other clones of Type-1 cells (1B11, 2C10, 3D10, 4B7, 4F2) had very similar profiles, showing efficient virus replication with peaks of infection between 72–96 hours post infection with the maximum infectivity titers in the modest range of 5.5 and 6.4 log_10_/0.1 mL (TCID_50_) and titers of hemagglutination 1∶32–1∶64. The dome-forming cell line 3E11 (Type-3) was found as the most permissive trypsin-independent substrate for H1N1 replication and the only clone where the hemagglutination titers reached 1∶128.

The effect of trypsin on H1N1 virus growth kinetics in Type-1 cells was evaluated in two clones, 2F8 and 4F2, and was found to be considerable, increasing the infectivity titers in the range of 1 log_10_ in the corresponding cell line and about a 2-fold increase in hemagglutination titers. In the clone 2F8, virus replication even in the presence of trypsin was still at a level significantly below average observed for other cell clones (Fig. S2).

### Characterization of the HA1/HA2 Cleavage Profile

**Figure 8:**
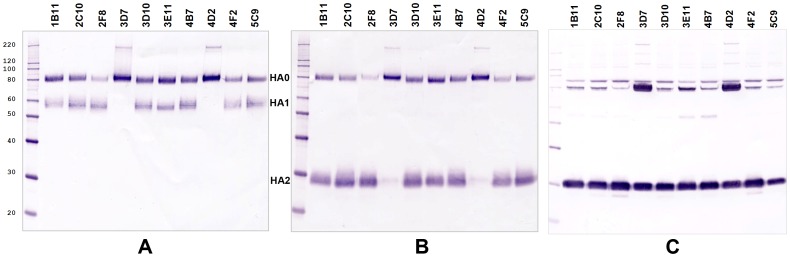
HA1/HA2 cleavage profile. The HA1/HA2 cleavage profile of the HA of A/Brisbane/59/2007 IVR-148 (H1N1, seasonal) and B/Hubei-Wujiagang/158/2009 grown in the corresponding cell clones (without addition of trypsin to the culture media) was evaluated by SDS-PAGE (reducing conditions) followed by Western blot with rabbit polyclonal antibodies specific to HA1 or HA2 portions of the viral hemagglutinin. Panel A: Western blot of H1N1 with anti-HA1 antibodies. Panel B: Western blot of H1N1 with anti-HA2 antibodies. Panel C: Western blot of B/Hubei with anti-HA2 antibodies. The protein bands corresponding to the non-cleaved HA0 migrate at the level of 80 kDa; bands representing HA1 and HA2 molecules migrate at the level of ∼58 kDa and 28–30 kDa, respectively.

The HA1/HA2 cleavage profile of the hemagglutinin (HA) of viruses grown in each cell clone was evaluated by SDS-PAGE (under reducing conditions) followed by Western blot with anti-HA1 or anti-HA2 antibodies. Influenza A viruses represented by H1N1 virus strains (seasonal and H1N1pdm09) and one strain of influenza B virus were used for this purpose. In all Type-1 cell clones and in clone 3E11 (Type-3 cells), replication of the influenza A viruses (H1N1) was associated with proteolytic activation of the viral hemagglutinin; both bands, representing HA1 and HA2, were clearly identifiable in Western blots. In contrast, Type-2 clones 3D7 and 4D2 produced only HA0, the non-cleaved precursor. The HA1/HA2 cleavage profiles of IVR-148 (H1N1 seasonal) grown in different cell clones are presented in Fig. 8. Identical cleavage profiles were observed for two others H1N1 viruses, representing 2009-pandemic strains: A/California/7/2009 X-179A and A/Christchurch/16/2010 (data not shown). The cleavage profiles of HA of B/Hubei-Wujiagang/158/2009 grown in each of the clones are shown in Fig. 8C. Apart from influenza A viruses (H1N1), the cleavage of HA of influenza B virus was observed in all tested cell clones. However, in the 3D7 and 4D2 clones (Type-2 cells), the HA1/HA2 processing was substantially diminished than in any other clone analyzed. Noteworthy is that virus samples even from the cell clones where HA1/HA2 cleavage was observed (Type-1 and Type-3 cells) always contained a significant proportion of the non-cleaved HA0, probably indicating that at some time in the infection cycle the cellular proteolytic pathway was altered by virus infection leaving some of the HA0 molecules unprocessed. This investigation revealed that selected MDCK clones differed in their ability for proteolytic activation of viral hemagglutinin. The Type-1 and Type-3 cells produced proteolytically activated HA1/HA2 in all instances, whereas in the Type-2 cells, this function was significantly restricted (for influenza B virus) or eliminated (H1N1, influenza A virus).

The data obtained in this study are summarized in Table 1 and Table S1.

## Discussion

The heterogeneity of the original MDCK cell line is well documented, and cell strains and clones with different morphology, electro-physiological, and biochemical properties have been described [6], [7], [24], [38], [49], [51]–[53], [56]–[58], [60], [61], [63], [71], [76], [78], [79]. In this study, cloned cell lines, each of which originated from cells randomly selected by liming dilution of the original MDCK cell line (ATCC-CCL-34, NBL-2; +20 passages), were investigated and characterized for their capacity to support replication of different influenza viruses and applicability for specific virological tests.

Based on cellular morphology, three distinct cell types were identified. The characteristic feature of cells of Type-1 morphotype is a gentle monolayer of flattened polymorphic cells with almost indistinguishable cell boundaries and clearly visible nuclei. Extracellular liquid-filled structures (domes) were not observed in these cell lines. However, spontaneous formation of the giant multinucleated cells was frequently observed, though without strict periodicity or obvious cause. Appearance of the giant cells in the MDCK cultures had also been described by other investigators [24], [80], and it was determined that they represented end-stage post-mitotic cells [24].

The cells of Type-2 morphology are characterized by a relative uniformity in size, angled polygonal perimeter, and well-outlined intercellular space with rough, phase-bright, and often grainy appearance under light microscope. The transparency of the monolayer is diminished, in comparison with Type-1 cells, and the nucleus is almost indistinguishable. Cells of this type form a monolayer with a very characteristic mosaic pattern, clear of extracellular liquid-filled formations.

It had already been reported by other investigators that two types of cells with the morphological properties very similar to those described in this study could be isolated by cloning of the original MDCK cell line. Therefore, to simplify the data analysis and interpretation, we adopted the same nomenclature (Type-1 and Type-2) for the cell lines showing the corresponding morphological similarities with the cell lines described earlier [7], [24], [48], [49], [51], [52], [54], [56]–[58], [61], [77]. Both morphotypes had been extensively characterized for a variety of electro-physiological and biochemical parameters that are used mostly as an *in vitro* model for studying renal differentiated epithelial cells. It was shown using electron microscopy that the Type-1 cells are flattened (with cell height about 1–3.3 µm) and have a very tight intercellular connections, whereas Type-2 cells had cuboid or columnal shape, significantly taller (with cell height ranging between 6–10 µm) and with a very sparse intercellular junction and dilated intercellular space [24], [49], [50], [53], [57], [61]. The observed difference in intercellular connections correlated with the transepithelial electrical resistance - the characteristic marker of permeability of confluent monolayer. The monolayer of tightly interconnected Type-1 cells was characterized by high resistance, whereas the monolayer of the Type-2 cells showed very low resistance [24], [48], [49], [51], [52], [54], [61].

In addition to these two easily distinguishable Type-1 and Type-2 cell morphologies, one clone investigated in this study was segregated into a separate group, designated in this study as Type-3. The characteristic feature of this cell line was its ability to form numerous extracellular liquid-filled rounded structures (domes) under standard conditions of cell maintenance and cultivation. The liquid-filled structures formed by MDCK cells were first described in 1969 and named “domes” [65], and the phenomenon was well investigated as a model of epithelial cell function. It was determined that dome formation is sustained by tight intercellular junctions and associated with a unidirectional ion transport into a lumen formed between a solid substratum and the basolateral cell surface, resulting in change of the osmotic pressure, followed by the flow of water into the lumen and inflation of blister-like domes [54], [55], [66], [81]–[84]. The published material regarding which type of cell (Type-1 or Type-2) has a tendency of dome formation is inconsistent, especially taking into account the fact that in many cases dome formation was investigated using specific chemical stimulants, hormones, or special culture medium composition affecting the salt balance [21], [24], [48], [53], [55], [57], [60], [66], [83]–[88]. It was also reported that cryoprotective agents, such as DMSO and DMF, might induce dome formation [55], [66], [84]. In addition, it was shown that the dome formation could be reversed under conditions of mechanical stimulation (shear stress by apical flow) [81]. One of the recent reports provided additional evidence that the cyst formation induced by specific chemical agents is a property of cells with Type-1 phenotype [85]. There are still no sufficient data to conclude with confidence whether the dome-forming cells represent a separate independent genetically determined type of cells or the cells that are undergoing differentiation in response to the changing environment. Microscopic monitoring of the dome-forming clone 3E11 showed that its morphology is very similar to that of the Type-2 cells (mosaic monolayer of angled polygonal cells with bright-phase borders, hardly visible nucleus). At the same time, other properties, such as the level of expression of PNA ligand and the ability to support multicycle replication of influenza A viruses without exogenous trypsin (due to efficient HA1/HA2 cleavage) make this clone closer to Type-1 cells. Taking into account all evaluated phenotypic properties, this cell clone could not be classified as an authentic representative of Type-1 or Type-2 cells and was segregated as a representative of another cell type (Type-3; “dome-forming” cells).

Regardless of the morphological classification, all cell clones showed susceptibility to infection by various influenza viruses, including H1N1 (seasonal and H1N1pdm09), H3N2, H5N1, and influenza B virus, indicating that all of them have surface receptors (terminally sialylated glycans) in an amount that ensure virus attachment and initiation of infection. Analysis of all clones by lectins that selectively bind sialic acids with either α2–3 or α2–6 glycosidic linkage (lectins MAA and SNA, respectively), as well as probing of cell clones by influenza B virus variants with α2–3 or α2–6 binding preferences, qualitatively showed that all clones have both types of receptors. At the same time, analysis of the expression of PNA-specific ligand clearly revealed differences between Type-1 and Type-2 cells, suggesting that the spectrum of the surface carbohydrates may vary significantly depending on cell type. These results are in accord with the published data, which showed that cells of both morphotypes (Type-1 and Type-2) have a spectrum of similar and identical glycans, including glycolipids and phospholipids, with the majority of the gangliosides being represented by GM_3_ (terminally sialylated via α2–3 linkage) [52], [58], [89]. However, significant differences were observed in expression of glycosphingolipids. Thus, Type-1 cells were found to be rich with fucolipids, deficient in expression of the Forssman glycolipids [52], [58] and PNA-negative [51], [53], whereas Type-2 cells express sulfatide (galactosylceramid-3-sulphate) [52], [90] and globo series, including globoside and Forssman antigen [52], [58] and were identified as PNA-positive [51], [53], [77]. The presence of the sialylated glycans with both α2–3 and α2–6 types of glycosidic linkage on the surface of the original non-cloned MDCK cells has already been reported [32], [91]–[96]. The data presented in this study expand this knowledge further, demonstrating that cells of different morphotypes isolated from the MDCK cell line still express sufficient amounts of sialylated glycans with both types of glycosidic linkage. The evaluation of the expression of non-sialylated PNA-specific carbohydrates could be a useful tool for typing and sorting of the MDCK clones.

The data presented in this study demonstrated that not all cell clones, even those of the same morphotype, are similarly permissive for the replication of influenza viruses. Influenza B virus was found to be the least demanding to the cell properties but still showed some variability in plaque size phenotype and rate of virus accumulation in the growth kinetics experiments. In contrast, the influenza A viruses, especially H1N1 and H3N2, and to a lesser degree the H5N1, showed explicit discrimination. Thus, none of the influenza A viruses was capable of multicycle replication in Type-2 cells (3D7, 4D2) in the absence of exogenous trypsin. The deficiency of those cell clones to produce proteolytically activated hemagglutinin (cleaved between HA1/HA2) was confirmed by Western blot analysis of various H1N1 strains, where only non-processed HA0 molecules were detected. Interestingly, the same cell clones were able to produce cleaved HA of influenza B virus, though at a significantly lesser extent if compared with the cleavage in all other tested clones. Moreover, in these Type-2 cells influenza B viruses produced biggest plaques. On the other hand, the supplementation of the culture medium with trypsin made these Type-2 cell lines the most productive substrates to propagate influenza A viruses, as was demonstrated by the virus growth kinetic experiments and plaque assays, where the positive response to trypsin addition was the most conspicuous. Whether the observed differences in HA cleavage profile of influenza A and B viruses are associated with predominant expression of particular types of cellular proteases, variations in glycosylation pathways, or indicative of the specifics of viral hemagglutinin, is not clear and requires additional studies. It may indicate evolutionary adaptation of influenza B viruses for the cells of mammalian origin.

Influenza A viruses showed individual preferences for the specific cell lines. Thus, clone 4F2 was identified as a good substrate for H1N1 and H5N1, but not for H3N2 strains where the plaque formation by this virus was significantly inhibited even in the presence of trypsin. Addition of trypsin usually led to a considerable increase of plaque size. Clone 2F8 was least permissive for plaque formation by any viruses even in the presence of the trypsin. In addition, clone 5C9 was found to be impractical for plaque assay due to its extreme sensitivity to the media environment, being unable to survive in the agar-containing media. Kinetics experiments with influenza A virus (H1N1) also showed the inferiority of 2F8 and 5C9, to other clones. Evaluation of the influence of the exogenous trypsin on virus growth kinetics in Type-1 cells was performed with two clones, 2F8 and 4F2, and showed that presence of trypsin in the media had some positive (though modest) impact on the dynamic of virus replication, and was associated with increase of infectivity titers for approximately 1.0 log_10_ (and 2–4-fold increase of HA titers; data not shown) in each cell line, respectively, but still requiring approximately the same period (∼72 hours) to reach the peak. However, virus replication in 2F8 was still at a low level even with trypsin.

The observations accumulated in this study clearly indicate that cell clones even from the same morphological type (Type-1) still may have other significant differences and physiological properties that would affect replication of influenza viruses. It was shown by other investigators that different morphotypes of MDCK cells vary in a number of physiological properties, including intracellular pH and direction of ion transport and secretion [51], [63], [79], [97] that would be also associated with the observed variability of cell clones to support virus replication.

The data presented here also demonstrate that viruses of different subtypes have different requirements for efficient replication in MDCK clones. Influenza B virus was fit to replicate in all tested clones, including those that have a Type-2 morphology with reduced proteolytic capabilities (clones 3D7 and 4D2). The H5N1 strain was the least demanding among the tested influenza A viruses, whereas the H3N2 strain was the most sensitive to the properties of the substrate and trypsin supplementation.

Taken together, this study illustrates that the cell lines cloned from the original MDCK cells vary in morphology, carbohydrate representation, and ability to support efficient replication of influenza viruses representing different virus species or subtypes. Some of the described clones could be the cell lines of choice for specific research applications, such as plaque assay, virus propagation, or infectivity titration. In addition, clones with various HA1/HA2 proteolytic capabilities, producing either non-cleaved HA0 or cleaved HA1/HA2, may be used as another tool in studying the biosynthetic pathway of influenza hemagglutinin.

## Supporting Information

Figure S1 Plaque size phenotype of influenza A and B viruses in MDCK clones (−/+Trypsin). Photographs of plaques 96 hours post infection in the absence (T−) or presence (T+) of Trypsin in the overlaying agar media in the corresponding cell clones of the following virus strains: A/Brisbane/59/2007 IVR-148 (H1N1, seasonal); A/Uruguay/716/2007 X-175C (H3N2), rg-A/Vietnam/1203/2004 (H5N1, low-pathogenic), B/Hubei-Wujiagang/158/2009, and B/Victoria/504/2000.
(TIF)

Figure S2 Effect of exogenous trypsin on the growth kinetics of IVR-148 (H1N1) in MDCK clones. Culture of the corresponding MDCK clones (confluent cell monolayer) were infected by A/Brisbane/59/2007 IVR-148 (H1N1, seasonal) at MOI 0.000001 and incubated at 33°C until complete destruction of the monolayer associated with cytopathogenic effect (CPE). Trypsin was added to the corresponding flasks 4 hours post infection, at a final concentration 1.0 µg/ml of media. Open squares represent virus growth without trypsin in the culture media; filled (red) squares represent virus growth in the presence of exogenous trypsin. Accumulation of the virus in the culture was determined by infectivity titration (TCID_50_, log_10_/0.1 ml) of the samples of the cell culture media collected every 24 hours post infection.
(TIF)

Figure S3 Mean Fluorescence Intensity of the cell-bound FITC-labeled PNA. The level of cell surface expression of PNA-specific glycans was evaluated by flow cytometry using FITC-labeled PNA. Data shows mean fluorescence intensity from one representative experiment.
(TIF)

Table S1 Characterization of MDCK clones as substrates for H3N2 and H5N1 influenza A viruses. Efficiency of cell clones to support replication of H3N2 and H5N1 viruses was evaluated by plaque assay with and without trypsin in the overlaying agar-containing media.
(DOC)
