# Supplementary figures and images for: Heterogeneity of the MDCK Cell Line and Its Applicability for Influenza Virus Research
Source: PLoS One. 2013 Sep 13;8(9):e75014. doi: 10.1371/journal.pone.0075014 (PMC3772841; doi:10.1371/journal.pone.0075014)

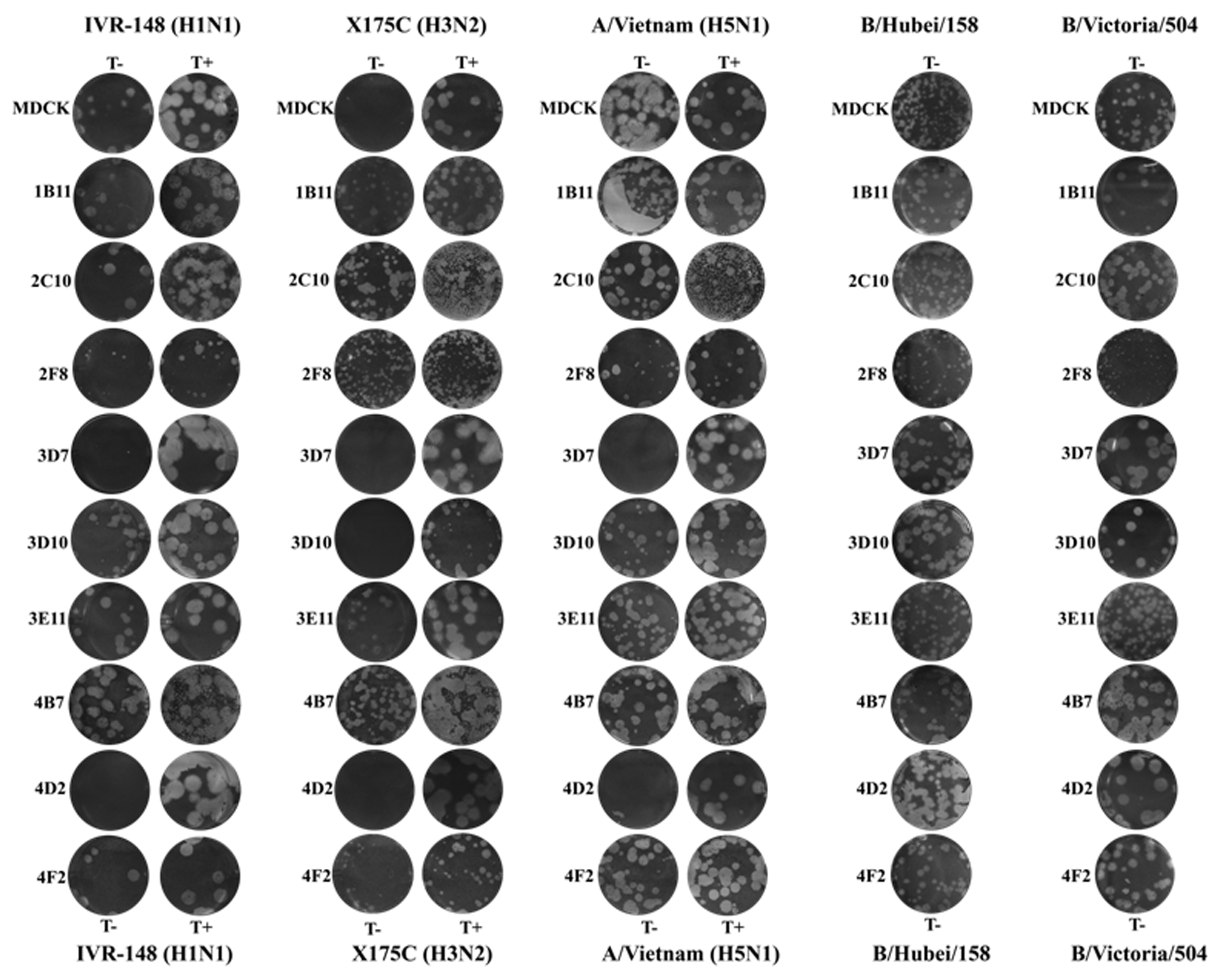

Supplement: Figure S1 — Plaque size phenotype of influenza A and B viruses in MDCK clones (−/+Trypsin). Photographs of plaques 96 hours post infection in the absence (T−) or presence (T+) of Trypsin in the overlaying agar media in the corresponding cell clones of the following virus strains: A/Brisbane/59/2007 IVR-148 (H1N1, seasonal); A/Uruguay/716/2007 X-175C (H3N2), rg-A/Vietnam/1203/2004 (H5N1, low-pathogenic), B/Hubei-Wujiagang/158/2009, and B/Victoria/504/2000. (TIF) [file pone.0075014.s001.tif]

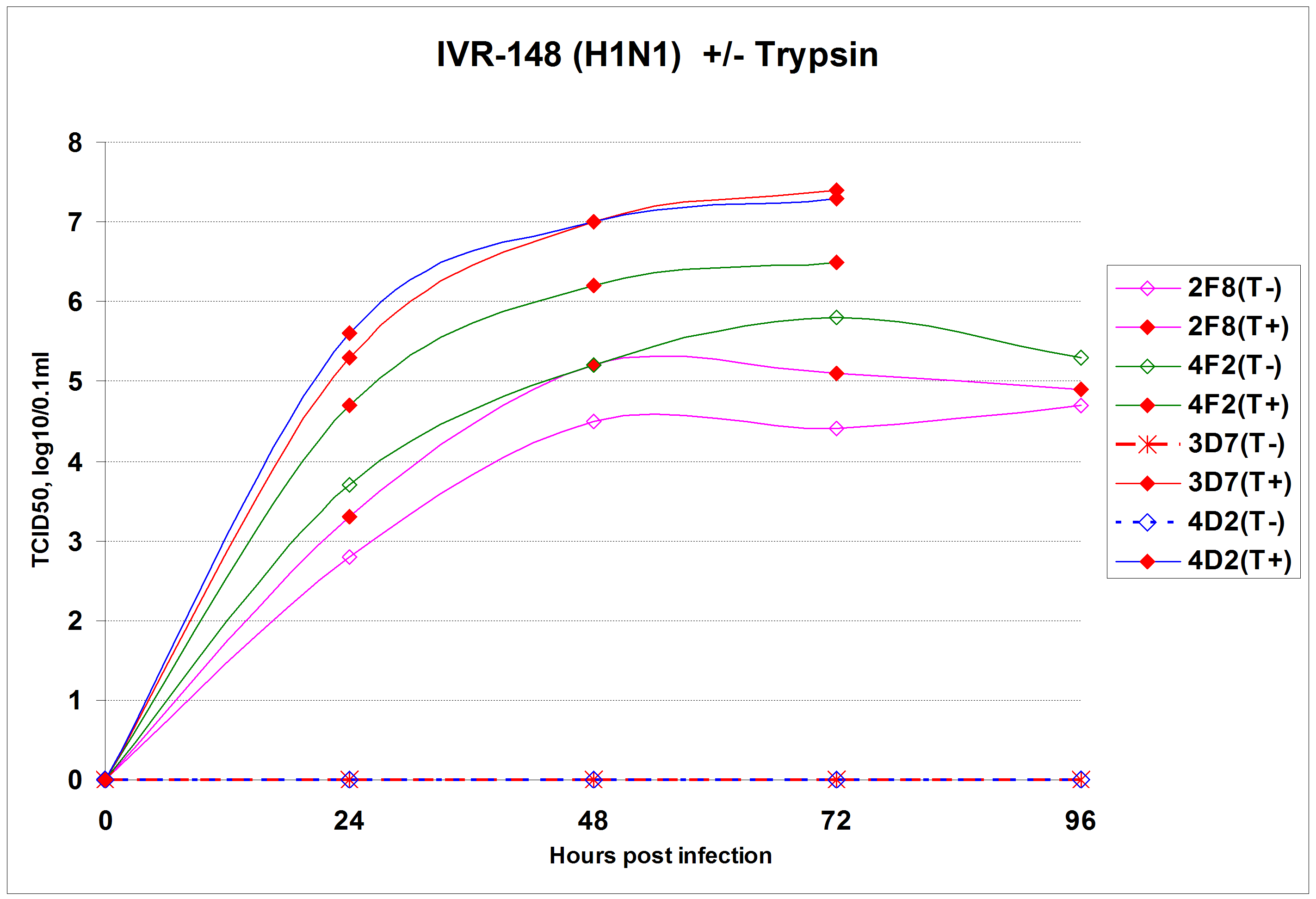

Supplement: Figure S2 — Effect of exogenous trypsin on the growth kinetics of IVR-148 (H1N1) in MDCK clones. Culture of the corresponding MDCK clones (confluent cell monolayer) were infected by A/Brisbane/59/2007 IVR-148 (H1N1, seasonal) at MOI 0.000001 and incubated at 33°C until complete destruction of the monolayer associated with cytopathogenic effect (CPE). Trypsin was added to the corresponding flasks 4 hours post infection, at a final concentration 1.0 µg/ml of media. Open squares represent virus growth without trypsin in the culture media; filled (red) squares represent virus growth in the presence of exogenous trypsin. Accumulation of the virus in the culture was determined by infectivity titration (TCID50, log10/0.1 ml) of the samples of the cell culture media collected every 24 hours post infection. (TIF) [file pone.0075014.s002.tif]

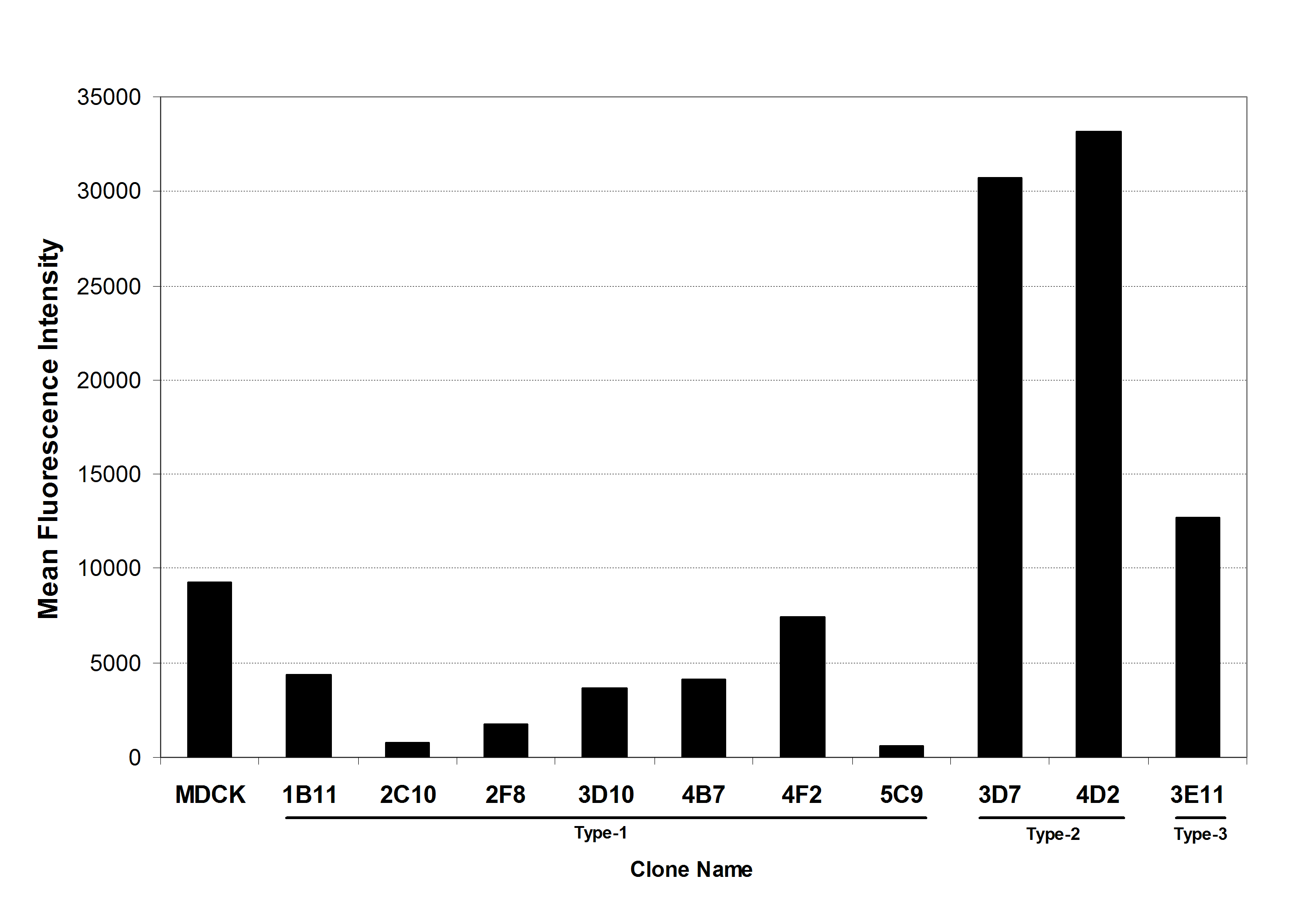

Supplement: Figure S3 — Mean Fluorescence Intensity of the cell-bound FITC-labeled PNA. The level of cell surface expression of PNA-specific glycans was evaluated by flow cytometry using FITC-labeled PNA. Data shows mean fluorescence intensity from one representative experiment. (TIF) [file pone.0075014.s003.tif]
