# Supplementary material for: Heterogeneity of the MDCK Cell Line and Its Applicability for Influenza Virus Research
Source: PLoS One. 2013 Sep 13;8(9):e75014. doi: 10.1371/journal.pone.0075014 (PMC3772841; doi:10.1371/journal.pone.0075014)
Supplement: Table S1 — Characterization of MDCK clones as substrates for H3N2 and H5N1 influenza A viruses. Efficiency of cell clones to support replication of H3N2 and H5N1 viruses was evaluated by plaque assay with and without trypsin in the overlaying agar-containing media. (DOC) [file pone.0075014.s004.doc]

**TABLE S1. Characterization of MDCK clones as substrates for H3N2 and H5N1 influenza A viruses.**

|  |  | Support virus replication without trypsin* | | Plaque size, mm  Mean ± SD | | | |
| --- | --- | --- | --- | --- | --- | --- | --- |
| MDCK Clone | Morphotype | H3N2  X-175C | H5N1  A/Viet | H3N2 Trypsin  (-) | H3N2  Trypsin  (+) | H5N1 Trypsin  (-) | H5N1  Trypsin  (+) |
| 1B11 | I | + | + | 1.2±0.4 | 2.4±0.7 | 1.3±0.4 | 2.0±0.6 |
| 2C10 | I | + | + | 1.3±0.5 | 1.1±0.2 | 2.1±0.7 | 1.6±0.3 |
| 2F8 | I | + | + | 0.4±0.1 | 0.5±0.2 | 1.2±0.6 | 1.4±0.5 |
| 3D10 | I | + | + | 0 | 1.6±0.6 | 1.5±0.6 | 2.8±0.5 |
| 4B7 | I | + | + | 1.6±0.5 | 3.0±0.3 | 2.1±0.6 | 2.0±0.9 |
| 4F2 | I | + | + | 0.5±0.2 | 0.9±0.5 | 2.2±0.6 | 0.4±0.1 |
| 5C9 | I | + | + | *No data* | *No data* | *No data* | *No data* |
| 3D7 | II | – | – | 0 | 3.2±0.4 | 0 | 2.9±0.4 |
| 4D2 | II | – | – | 0 | 3.6±0.5 | 0 | 3.1±0.5 |
| 3E11 | III | + | + | 1.4±0.4 | 2.7±0.5 | 1.3±0.4 | 2.1±0.3 |
| MDCK  (parent) | *mix* | + | + | 0.7±0.3 | 2.3±0.3 | 2.5±0.5 | 2.5±0.6 |

*Based on the results from plaque assay.
